# Silane Coupling Agent Modifies the Mechanical Properties of a Chitosan Microfiber

**DOI:** 10.3390/molecules25225292

**Published:** 2020-11-13

**Authors:** Yuki Shirosaki, Toshinobu Okabayashi, Saki Yasutomi

**Affiliations:** Faculty of Engineering, Kyushu Institute of Technology, 1-1 Sensui-cho, Tobata-ku, Kitakyushu, Fukuoka 804-8550, Japan; o109011t@mail.kyutech.jp (T.O.); s-yasutomi@che.kyutech.ac.jp (S.Y.)

**Keywords:** chitosan, silane coupling agent, microfiber, crosslinking, mechanical strength

## Abstract

Chitosan microfibers are widely used in medical applications because they have favorable inherent properties. However, their mechanical properties require further improvement. In the present study, a trimethoxysilane aldehyde (TMSA) crosslinking agent was added to chitosan microfibers to improve their tensile strength. The chitosan microfibers were prepared using a coagulation method. The tensile strength of the chitosan microfibers was improved by crosslinking them with TMSA, even when only a small amount was used (less than 1%). TMSA did not change the orientation of the chitosan molecules. Furthermore, aldehyde derived from TMSA did not remain, and siloxane units were formed in the microfibers.

## 1. Introduction

Microfiber-based materials are widely used in medical applications, such as sutures, textiles, and scaffolds [1,2,3]. Natural polymers are good candidates for the preparation of microfibers because they are biocompatible, non-toxic, biodegradable, and have low immunogenicity. Chitin and chitosan are candidates for biofibers, and their inherent properties have been reported [4]. Chitosan fibers are easier to prepare than chitin fibers because they are soluble in dilute acids.

Chitosan is often crosslinked with reagents, such as glutaraldehyde and epoxy compounds, to improve its mechanical properties and control its biodegradability [4,5,6]. However, such compounds are highly cytotoxic [7] and reduce the biocompatibility of chitosan. Shirosaki et al. already used the silane coupling agent γ-glycidoxypropyltrimethoxysilane (GPTMS) to crosslink chitosan and demonstrated improvements in the mechanical properties of a chitosan membrane [8]. The epoxide groups of GPTMS can react with the amino groups of chitosan, and each GPTMS molecule polycondenses to form −Si−O−Si− networks. The crystalline properties of chitin and chitosan are also important for their mechanical characteristics. The addition of GPTMS can disturb the orientation of the crystalline domains of chitosan [8,9], reducing its mechanical strength.

**Figure 1:**
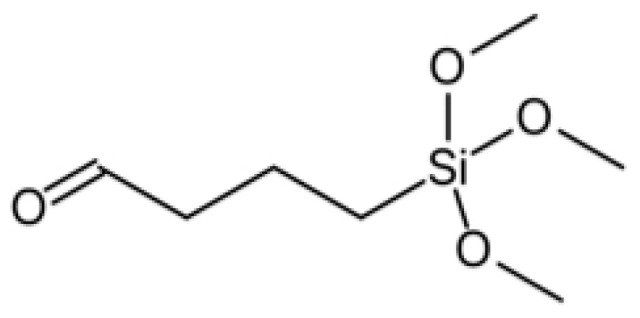
Trimethoxysilane aldehyde (TMSA).

**Figure 2:**
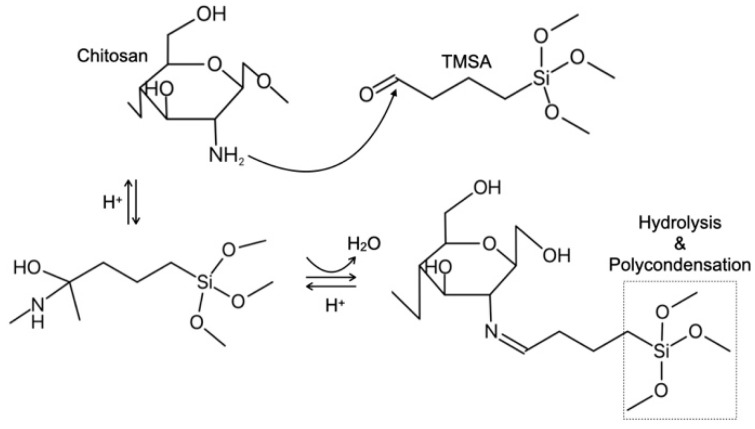
Expected reaction between chitosan and TMSA forming a Schiff base.

In the present study, we used trimethoxysilane aldehyde (TMSA) shown in Figure 1 as a crosslinked agent to improve the tensile strength of the chitosan microfibers. It is to be expected that, compared with GPTMS, a smaller amount of TMSA can effectively react with the amino groups in chitosan owing to crosslinking by the aldehyde groups and the polycondensation of the silanol groups derived from the three methoxysilane groups of TMSA. The reaction between the amino groups of chitosan and the aldehyde groups of glutaraldehyde results in the formation of a Schiff base [10]. The aldehyde groups of TMSA can react as shown in Figure 2.

## 2. Results

### 2.1. Structural Characterization

**Figure 3:**
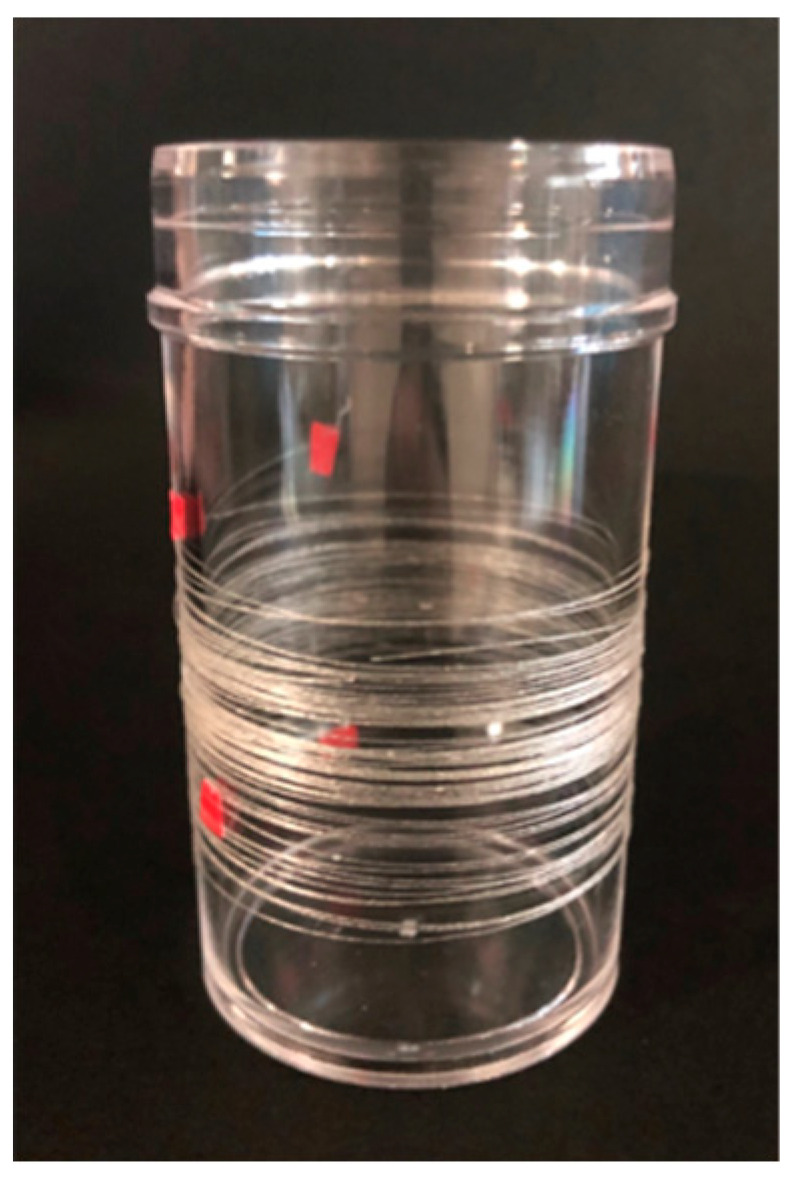
Photograph of the chitosan−TMSA microfiber (ChTMSA001).

**Figure 4:**
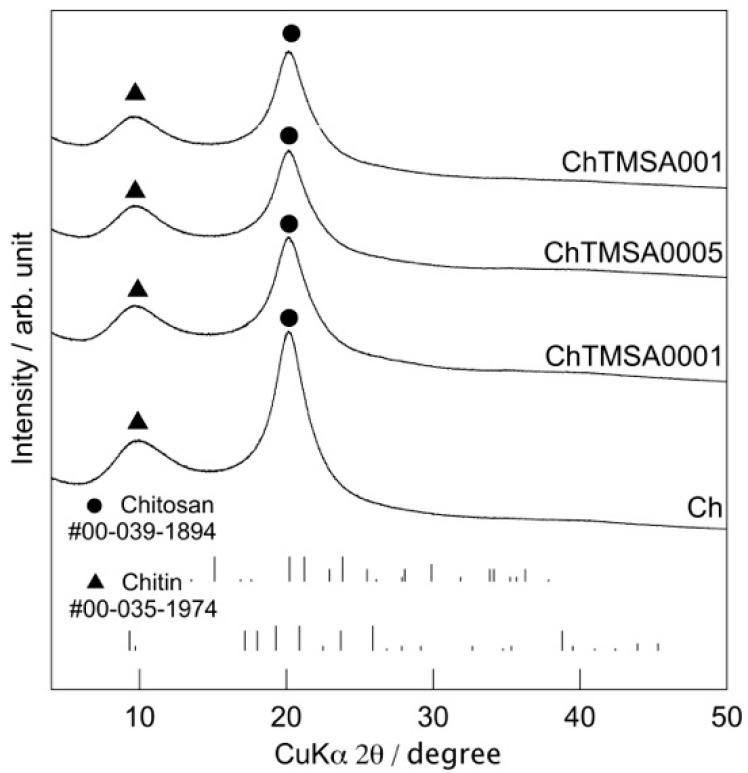
XRD patterns of the chitosan−TMSA microfibers.

The fibers were white and flexible, and the diameter was around 200 µm, regardless of the amount of TMSA added (Figure 3). Figure 4 shows the X-ray diffraction (XRD) patterns of the fibers. Only the peaks attributable to chitosan (2θ = 20°, PDF #00-039-1894) and chitin (2θ = 10°, PDF #00-035-1974) were detected. The half width at 2θ = 20° is shown in Table 1. The half width value did not change after the addition of TMSA.

**Figure 5:**
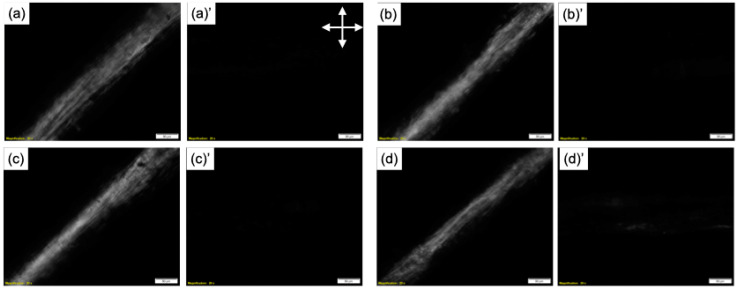
Optical microscope images of the chitosan−TMSA microfibers obtained with crossed nicols at 45° (**a**–**d**) and 90° (**a**)′–(**d**)′. (**a**) and (**a**)′ Ch; (**b**) and (**b**)′ ChTMSA0001; (**c**) and (**c**)′ ChTMSA0005; and (**d**) and (**d**)′ ChTMSA001. The arrows indicate the direction of the nicols.

**Figure 6:**
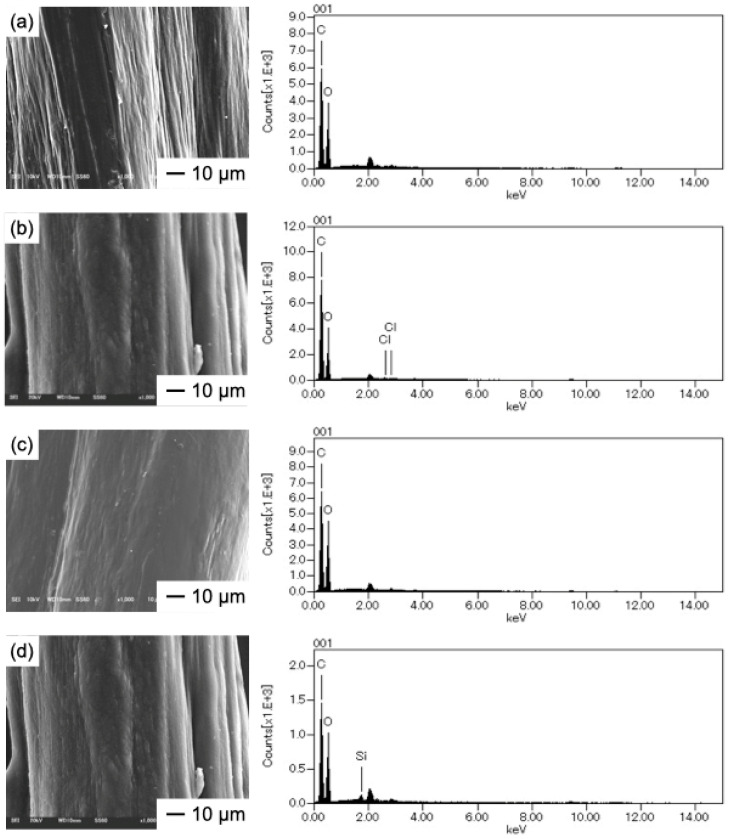
SEM images and EDS spectra of the chitosan−TMSA microfibers. (**a**) Ch; (**b**) ChTMSA0001; (**c**) ChTMSA0005; and (**d**) ChTMSA001.

The intensity of birefringence in the microscope images obtained using crossed nicols (Figure 5) confirms the orientation of the chitosan molecules in the fibers. Figure 6 shows the scanning electron microscope (SEM) images and energy-dispersive X-ray spectroscopy (EDS) spectra of the fibers. The fibers had flattened surfaces and no deposits were discernable. Only fiber ChTMSA001 had silicon on its surface. The results of the ninhydrin test are shown in Table 2. The number of remaining amino groups decreased as the amount of TMSA increased, and fibers ChTMSA0001, ChTMSA0005, and ChTMSA001 had 102 ± 4%, 92 ± 2%, and 76 ± 1% amino groups, respectively.

**Figure 7:**
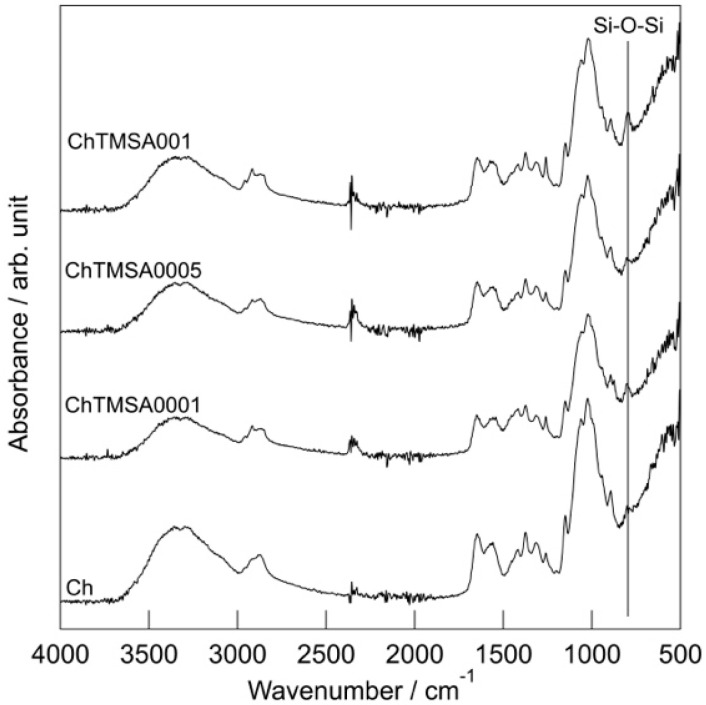
FT-IR spectra of the chitosan−TMSA microfibers.

Figure 7 shows the Fourier-transform infrared spectroscopy (FT-IR) spectra of the fibers. The characteristic bands of chitosan were detected in the spectra of all the fibers, as demonstrated in a previous paper [11]. Bands attributable to stretching in amide II (NH_2_ str.) were detected at approximately 1563 cm^−1^, and their intensity decreased after TMSA was added. The peaks in the spectra of the ChTMSA fibers at approximately 800 cm^−1^ can be attributed to symmetrical Si−O−Si stretching vibrations [12,13,14]. C=O derived from TMSA was not detected in the fibers.

### 2.2. Mechanical Strength

**Figure 8:**
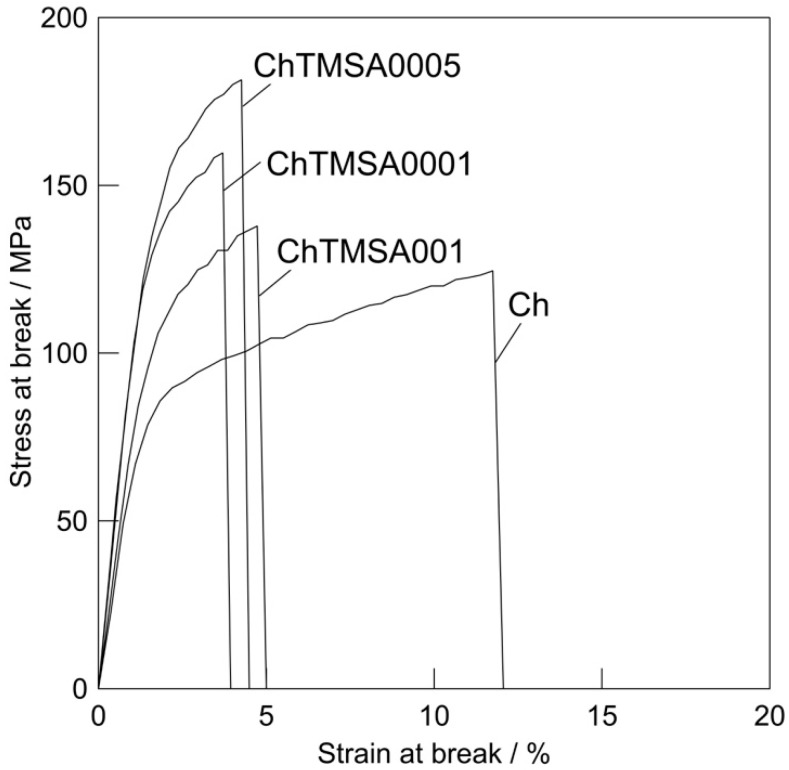
Stress–strain curves of the chitosan−TMSA microfibers.

**Figure 9:**
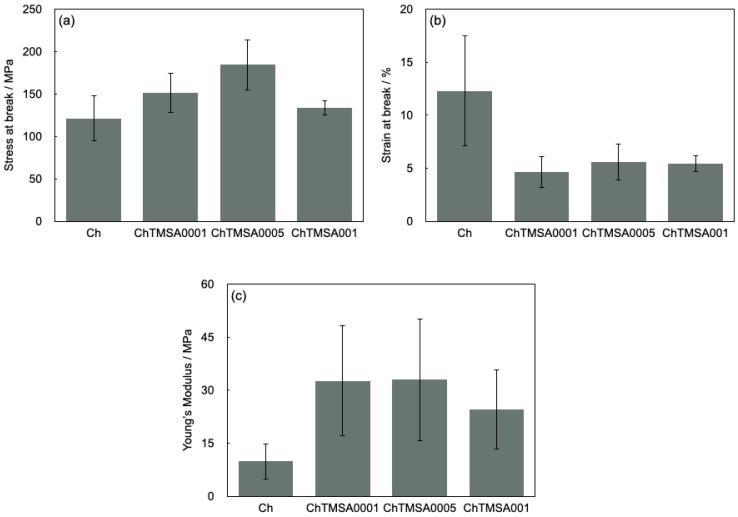
Maximum tensile strength values (**a**), maximum strain values (**b**), and Young’s modulus of the chitosan−TMSA microfibers (**c**). Both sets of values were obtained at the breaking point.

The results of the tensile strength tests are shown in Figure 8 and Figure 9. The maximum tensile strengths of Ch, ChTMSA0001, ChTMSA0005, and ChTMSA001 were 121.3 ± 26.7, 151.0 ± 23.5, 184.5 ± 30.0, and 133.8 ± 8.9 MPa, respectively (i.e., ChTMSA0005 had the highest value). The maximum strain values of Ch, ChTMSA0001, ChTMSA0005, and ChTMSA001 were 12.3 ± 5.2, 4.6 ± 1.5, 5.6 ± 1.7, and 5.4 ± 0.8%, respectively (i.e., the ChTMSA fibers had lower maximum strain values than Ch). The Young’s modulus values of Ch, ChTMSA0001, ChTMSA0005, and ChTMSA001 were 9.9 ± 5.1, 32.6 ± 15.7, 32.9 ± 17.3, and 24.6 ± 11.3 MPa, respectively (i.e., the ChTMSA fibers had higher Young’s modulus values than Ch).

## 3. Discussion

**Figure 10:**
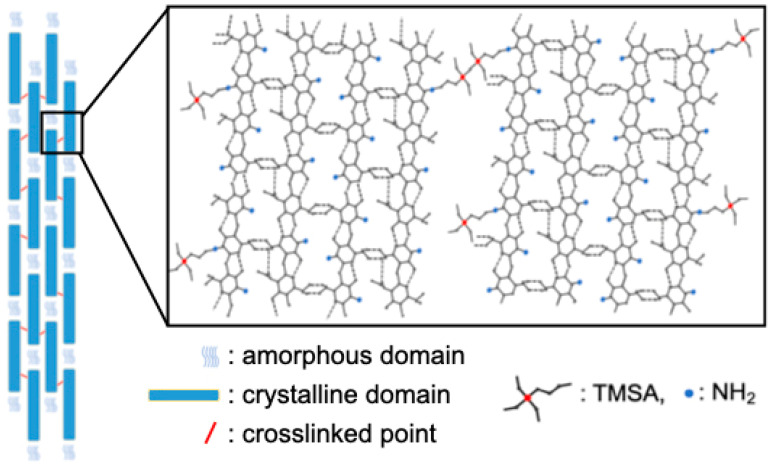
Structural model of the chitosan−TMSA (ChTMSA001) fiber.

Generally, chitosan molecules have crystalline and amorphous domains derived from the original structures of the chitin nanofibrils [15,16]. The XRD results reveal that the crystallinity of the chitosan molecules was retained even after the addition of TMSA. This suggests that the crystalline chitosan domains were not affected by crosslinking with TMSA. The ninhydrin tests insisted that the number of crosslinked amino groups was too small an amount even in ChTMSA001. FT-IR and the results of the ninhydrin tests demonstrated the existence of crosslinking between chitosan and TMSA. In the present study, the pH of the chitosan solution was approximately 4.0. Therefore, the amino groups probably formed −NH_3_^+^, thereby decreasing the nucleophilicity of the nitrogen atoms and the extent of the Schiff base reaction following mixing with TMSA [10]. However, the remaining TMSA was able to react completely with the amino groups of chitosan during washing with NaOH solution. According to the ninhydrin results, the crosslinked ration of ChTMSA001 was 20%. ChTMSA001 fibers refer to the addition of TMSA to produce 1% crosslinking of a chitosan unit. In addition to aldehyde groups, silanol groups interacted with the amino groups [9]. FT-IR showed symmetrical −Si−O−Si− stretching vibrations, which suggests that the −Si−OH groups formed by the hydrolysis of TMSA polycondensed in the chitosan fibers like silica [12,13,14]. However, the SEM images confirmed that there are no micro-sized deposits on the surfaces of the fibers. The EDS results also suggested that the addition of TMSA resulted in very little crosslinking within the chitosan molecules, in particular ChTMSA0001 and ChTMSA0005. Eventually, 19% of the amino groups in ChTMSA001 interacted with Si-OH and 1% were reacted with aldehyde. The orientation of the chitosan molecules in the fibers was also retained after the addition of TMSA. Figure 10 represents the expected molecular structure of the ChTMSA001 fiber according to the following characterization: (1) chitosan has crystalline and amorphous domains; (2) each bundle is oriented along the stretching direction; (3) just 1% of the amino groups in the fibers reacted with TMSA, and most of the TMSA reacted outside the bundles; and (4) the silanol groups derived from TMSA interacted with the chitosan molecules or polycondensed. The mechanical propertied of the fibers were improved by crosslinking. The increased Young’s modulus values insist that the interaction between chitosan become stronger and chitosan–TMSA fibers have more stiffness.

Silanol groups (−Si−OH) and siloxane networks (−Si−O−Si−) derived from GPTMS favored cell attachment and proliferation [8,9]. In the present study, FT-IR indicated that no aldehyde groups remained after the crosslinking reaction and siloxane networks formed. It is to be hoped that TMSA in the fibers is non-toxic and that chitosan−TMSA microfibers can be used in medical applications.

## 4. Materials and Methods

### 4.1. Preparation of Chitosan Monofibers with Silane Coupling Agent

Chitosan powder (high-molecular weight, molecular weight = 310,000—375,000 Da, degree of acetylation (DA) = 76.0%; Sigma-Aldrich^®^, St. Louis, MO, USA) was dissolved into 0.2 M aqueous acetic acid and mixed using a planetary centrifuge (ARE-310, Thinky Corp., Tokyo, Japan) to obtain a homogeneous 3.5% (*w*/*v*) chitosan homogeneous solution. The appropriate amount of TMSA (UCT, Bristol, TN, USA) was stirred in dimethyl sulfoxide to be hydrolyzed at room temperature, and the hydrolyzed precursor sols were obtained. The obtained precursor sols were added to a 3.5% (*w*/*v*) chitosan solution, and the mixture was mixed in a planetary centrifuge for 20 min. Mono-fibers were produced using a coagulation method, as described in a previous paper [17]. After washing with ethanol and a 0.2 M aqueous solution of sodium hydroxide, the fibers were stretched by rolling at 30/45 rpm with two rollers. Finally, the stretched fibers were dried at room temperature in a desiccator. The starting material composition of the fiber and sample code of each fiber are presented in Table 3.

### 4.2. Characterization and Mechanical Properties of the Fibers

The crystal structures of the fibers were examined using an automated multipurpose X-ray diffractometer (CuKα, 45 kV, 200 mA, 0.01°/step; Rigaku smartLab, Rigaku, Tokyo, Japan).

**Figure 11:**
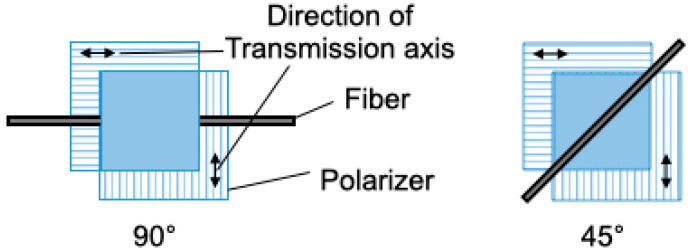
Examination of the molecular orientation of the fibers using crossed nicols. If the molecules were oriented, the image became dark at 90° and bright at 45°.

The orientation of the fibers was examined using an inverted microscope (IX73; Olympus Co., Tokyo, Japan) under crossed nicols. Each fiber was placed on a glass substrate and sandwiched between crossed nicols at 45° and 90° (Figure 11).

The surface morphology of each fiber was examined using an SEM (JMS-6010 PLUS/LA; JEOL, Tokyo, Japan) equipped with an EDX. Before examination, the fibers were coated with Pt/Pd to a thickness of approximately 20 nm (MSP-1S Magnetron Sputter; Vacuum Device Inc., Mito, Japan).

The molecular structures of the pulverized fibers were examined by FT-IR (FT/IR-6100; JASCO Co., Tokyo, Japan) using attenuated total reflectance at a resolution of 4 cm^−1^ and the accumulation of 100 scans.

The number of amino groups remaining in the fibers after crosslinking was evaluated by a ninhydrin assay [8]. The pulverized fibers (0.02 g) were suspended in 4 mL of ninhydrin solution (Ninhydrin Kit, L-8500 Set; Wako Chemicals, Osaka, Japan) and incubated at 80 °C for 40 min while shaking at 100 rpm. After cooling to room temperature, the optical densities of the sample (A_sample_) and chitosan (A_ch_) solutions were recorded at 570 nm using a spectrophotometer (DS-11+/w; DeNovix, Wilmington, DC, USA). The ninhydrin test was performed with replicates (*n* = 3). The number of the remaining amino groups was calculated using the following Equation (1):

Remaining amino groups (%) = Asample/Ach × 100(1)

As described in a previous paper [17], the mechanical properties of the fibers were examined by determining their tensile strengths at 0.5 mm/s using a creep meter (RE2-3305C; YAMADEN Co., Ltd., Tokyo, Japan).

## 5. Conclusions

Flexible chitosan microfibers were prepared by crosslinking them with TMSA. Results of XRD analysis showed that addition of TMSA did not change the crystallinity. Crossed nicols images also showed the orientation of chitosan. TMSA (molar ratio = 0005/amino groups chitosan) crosslinked only 1% of chitosan, but effectively improved the tensile strength of the microfibers (maximum strength around 184.5 MPa) and increased their stiffness. These mechanical properties depend on the crosslinking between chitosan and TMSA, as well as the –Si−O−Si– bonds of TMSA, which was confirmed by FT-IR.

## Figures and Tables

**Table 1:** The half width values at 2θ = 20° from XRD patterns.

Ch	ChTMSA0001	ChTMSA0005	ChTMSA001
1.0	1.0	1.0	1.0

**Table 2:** Ninhydrin test results indicating the number of amino groups remaining in the chitosan−TMSA microfibers.

Ch	ChTMSA0001	ChTMSA0005	ChTMSA001
100	102 ± 4	92 ± 2	76 ± 1

**Table 3:** Starting composition (molar ratio) of the fibers and sample code of each fiber.

	Ch	ChTMSA0001	ChTMSA0005	ChTMSA001
TMSA/Chitosan	0	0.001	0.005	0.01
